# Transforming Treatment: The Impact of Bariatric Surgery on Oral Drug Absorption

**DOI:** 10.1111/obr.70044

**Published:** 2025-11-30

**Authors:** Danielle Wigg, Andrea Edginton, Matthew D. Jones, Nikoletta Fotaki

**Affiliations:** ^1^ Department of Life Sciences University of Bath Bath UK; ^2^ Department of Bariatric Surgery, Southmead Hospital North Bristol NHS Trust Bristol UK; ^3^ School of Pharmacy University of Waterloo Waterloo Ontario Canada

**Keywords:** bariatric surgery, drug, gastrointestinal, oral absorption, PBPK modeling, pharmacokinetics, Roux‐en‐Y

## Abstract

Obesity represents a significant threat to global public health, with an estimated 16% of adults worldwide (2022) being classified as people with obesity, with a body mass index of 30 or more. Bariatric surgery is regarded as the most effective treatment option for people with obesity, with the three main types of bariatric surgery being gastric bypass or Roux‐en‐Y gastric bypass, laparoscopic gastric band, and laparoscopic sleeve gastrectomy. Drug bioavailability after oral administration is affected by several factors including properties of the drug itself, formulation properties, and anatomical and physiological factors. Procedures such as gastric bypass and laparoscopic sleeve gastrectomy result in significant anatomical and physiological alterations thought to profoundly influence oral drug bioavailability postoperatively. Consequently, following bariatric surgery, there is a risk of subtherapeutic drug levels leading to treatment failure, or the risk of potential toxicity if levels are elevated. In this review, previously unexamined aspects such as the impact of the “very low‐calorie diet” (VLCD) initiated prior to surgery on anatomical parameters and subsequent pharmacokinetic changes, are explored. This review also highlights alterations in hepatic and renal volume that are expected to have a significant impact on renal and hepatic clearance. A clearer understanding of the effect of physiological alterations and weight loss on drug performance after surgery would support more evidence‐based medicines optimization in this frequently complex patient group.

## 1 Introduction

Obesity represents a significant threat to global public health, with an estimated 16% of adults worldwide (2022) being classified as people with obesity, with a body mass index (BMI) of 30 or more [1]. In England, obesity prevalence is considerably higher, with 29% of adults living with obesity [2]. The pathogenesis of obesity has been recently understood as not simply an imbalance between calories consumed and energy expended, with metabolic parameters and appetite regulation via gastric and peripheral nervous systems understood to play a significant role [3]. All‐cause mortality (including cancer and cardiovascular disease) is known to be greater in obesity, with life expectancy (from the age of 40) demonstrated to be 4.2 years shorter in men with obesity and 3.5 years shorter in women [4]. Bariatric surgery is regarded as the most effective treatment option for people with obesity, with the three main types of bariatric surgery being gastric bypass or Roux‐en‐Y gastric bypass (RYGB), laparoscopic gastric band (LAGB), and laparoscopic sleeve gastrectomy (LSG) [5, 6].

Polypharmacy is increasingly prevalent in the population of patients with obesity when compared with the general population, due to the complex nature of their comorbidities. In one study, 53.5% of people with obesity (*n* = 615) were prescribed more than four drugs compared with 37.9% of the population without obesity (*n* = 436) [7]. Selective serotonin reuptake inhibitors (SSRIs), serotonin‐norepinephrine reuptake inhibitors (SNRIs), and direct‐acting oral anticoagulants (DOACs) are among the most commonly prescribed drugs in this population [8, 9] and will be used to illustrate the effects of bariatric surgery on drug bioavailability throughout this review.

Drug bioavailability after oral administration is affected by several factors including properties of the drug itself (e.g., solubility, permeability, stability), properties of the formulation (e.g., type of formulation, release/dissolution rate) and anatomical and physiological factors (e.g., gastric pH, intestinal surface area, and gastrointestinal transit time) [10, 11, 12]. Procedures such as RYGB and LSG result in significant anatomical and physiological alterations thought to profoundly influence oral drug bioavailability postoperatively [8, 13]. Consequently, following bariatric surgery, there is a risk of subtherapeutic drug levels leading to treatment failure or the risk of potential toxicity if levels are elevated [14].

Key questions surrounding oral drug bioavailability following bariatric surgery however remain unexamined, with only a small number of published short‐term pharmacokinetic studies, unable to capture physiological changes over time [9, 15, 16]. Recent reviews have provided a systematic overview of only some of the physiological changes after bariatric surgery, with the focus principally on RYGB, with subsequent reviews focused on identifying changes in key pharmacokinetic parameters (e.g., *C*_max_, *T*_max_, and AUC) for specific drugs [9, 17, 18, 19, 20, 21, 22]. The drugs examined in previous reviews include omeprazole, caffeine, metoprolol, midazolam, venlafaxine, and duloxetine [9, 17, 18]. The objective of this review is to explore in greater depth the impact of bariatric surgery, both RYGB and LSG, on all key physiological and anatomical parameters and their subsequent pharmacokinetic impact, in the context of current clinical practice.

## 2 Types of Bariatric Surgery

**FIGURE 1:**
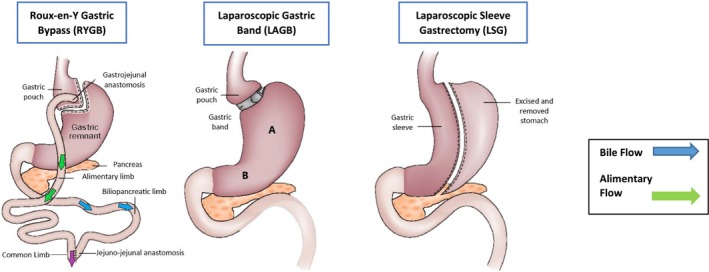
Diagrammatic representation of the two key bariatric procedures (modified from [23]).

Bariatric surgery is regarded as the most effective treatment option for people with obesity, with the two main types of bariatric surgery depicted in Figure 1: gastric bypass or RYGB and LSG [6, 23]. RYGB primarily consists of division of the upper portion of the stomach to create a small gastric pouch of 20–30 mL total capacity and the formation of a gastrojejunostomy, with resultant bypass of the proximal small intestine (between 50 and 150 cm) [24]. LSG consists of resection of the stomach fundus and gastric body, approximately two‐thirds of the total stomach volume, with the proximal small intestine remaining unaffected [25]. The mechanism by which these procedures produce weight loss is not fully understood; however numerous mechanisms including reduction in intake, hormone regulation, alteration of microbiota, and changes in the inflammatory process are thought to contribute to the overall decrease in total body weight [26] [27, 28].

As procedures such as laparoscopic banding have decreased in use due to suboptimal weight loss and a high complication rate, these procedures will not be discussed in this review [29].

## 3 Anatomical Parameters Before and After Bariatric Surgery

**FIGURE 2:**
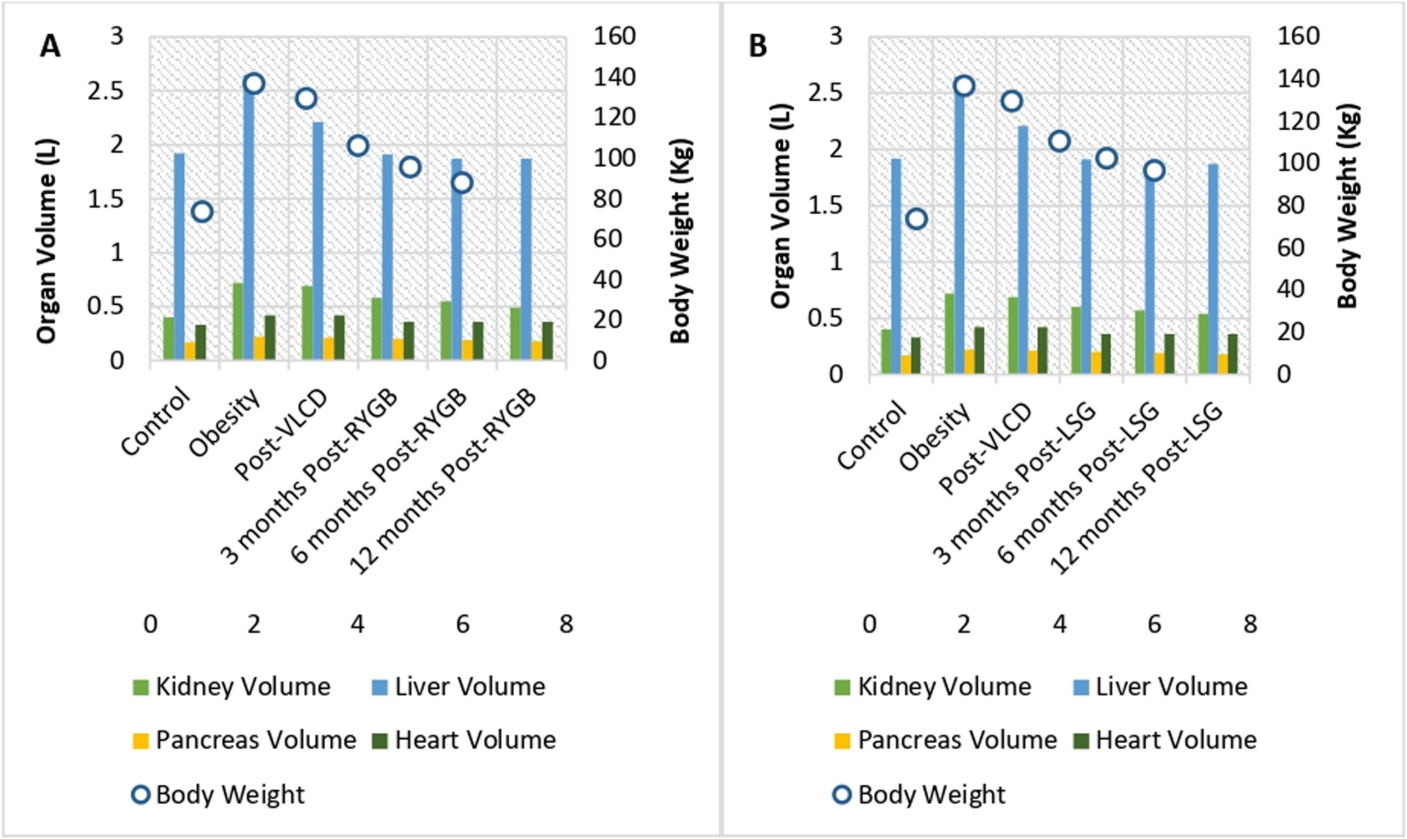
(A) Organ volume changes for female patients before and after RYGB when compared to the control population (BMI < 25 kg/m^2^). (B) Organ volume changes for female patients before and after LSG when compared to the control population (BMI < 25 kg/m^2^) [30, 31, 32, 33, 34, 35, 36, 37, 38, 41].

Obesity and the resultant weight loss following RYGB and LSG result in different organ sizes when compared to a control population with a BMI < 25 kg/m^2^ [30, 31, 32, 33, 34, 35, 36, 37, 38]. Figure 2A and B summarizes the changes in kidney, liver, heart, and pancreas sizes before and after RYGB and LSG.

Reference values in the “control” population (BMI < 25 kg/m^2^) were used to calculate kidney, pancreas, and liver sizes, with decreasing weight **(**Figure 2A,B) [30, 31, 32, 33, 34, 35, 36, 37, 38]. Figure 2 depicts calculated organ volume changes before and after RYGB (A) and before and after LSG (b) when compared to the control population [30, 31, 32, 33, 34, 35, 36, 37, 38]. With the bariatric surgery population being predominantly female, 80.5% (*n* = 3882), only female reference values were used [31]

### 3.1 Pancreas and Kidney Volumes

Pancreas and kidney volumes were calculated (by the authors), based on “control” values and the work by Grant et al., which quantified the relationship of BMI and organ volumes [32]. In this study, whole 3D volumes of solid organs were manually segmented for each participant who received a CT (computed tomography) scan (*n* = 750) [32]. The subjects were selected so as to exclude participants with known organ disease, with a range of BMIs from 30 to 50 kg/m^2^ [32]. The authors demonstrated that for every 5‐point increase in BMI from baseline, the volumes of the kidneys and pancreas increase by 11% (95% CI: 10%–12%) and 8% (95% CI: 7%–9%), respectively, and these values were used to estimate kidney and pancreas volumes at each respective time point [32].

### 3.2 Liver Volume

Prior to undergoing RYGB or LSG, it is common for patients to complete a very low‐calorie diet (VLCD), also known as a “liver shrinking diet”, to decrease liver size and thereby facilitate the intraoperative approach to the gastroesophageal junction [34, 35]. This aspect has been unexplored in previous studies and reviews exploring peri‐operative pharmacokinetic changes following bariatric surgery. However, a randomized controlled trial, exploring the effect of two preoperative weight‐loss diets (including VLCD) on liver volume has been conducted [35]. CT scans were performed without intravenous or oral contrast, with liver volume determined from the images taken between the lung bases and the third lumbar vertebra (L3) [35]. Changes in liver volume from the baseline “obesity” to “post‐VLCD” are shown in Figure 2A and B. Postoperative liver volume is based on a study that explored changes in liver volume at 3 and 6 months postoperatively, following RYGB and LSG [33]. The authors performed magnetic resonance imaging (MRI), with liver volume measurements made by outlining liver boundaries on each MRI slice [33].

With respect to the pharmacokinetic consequences associated with anatomical changes, alterations in liver and kidney volume would be expected to have a significant impact. Hepatic clearance is dependent on hepatic blood flow and enzymatic activity, with the presence of obesity significantly affecting liver size and hence hepatic blood flow [36, 37]. As SSRI/SNRIs are extensively hepatically metabolized, their clearance is likely to be affected by the altered liver size [37]. A positive correlation also exists between kidney volume and kidney function (measured creatinine clearance), with drugs such as DOACs that are reliant to some extent on renal clearance likely to be affected [38, 42].

### 3.3 Heart Volume

Heart volume is a key parameter, due to its influence on cardiac output, affecting drug absorption, distribution, metabolism, and elimination [43]. Increased cardiac output increases blood flow to the gastrointestinal tract (accelerating oral drug absorption), increases tissue perfusion (with associated more rapid distribution) and increases hepatic metabolism and renal clearance [43]. Two studies were used (by the authors) to determine heart volumes. Mean heart weights in female patients with obesity (median BMI 44.1 kg/m^2^; range 40–94.1 kg/m^2^) were obtained from a study detailing findings following post‐mortem examination [39]. Mean heart weights in relation to decreasing postoperative (RYGB and LSG) weight were determined from a large Swedish study (*n* = 27,645), which explored heart weight in medicolegal autopsies across a variety of body sizes, with an aim to determine reference values [40]. A value of 1.265 g/mL for myocardial tissue density was used to calculate heart volume (Figure 2A,B) [44].

## 4 Gastrointestinal Parameters Before and After Bariatric Surgery

### 4.1 Gastric Fluid Composition

#### 4.1.1 pH

Gastric pH influences drug solubility and dissolution, with pH determining the drug's ionization state [43]. The ionized state typically demonstrates increased drug solubility when compared to the unionized state [45]. Gastric pH in the general population is typically thought to range between 1 and 3.5 [10]. Several clinical studies have demonstrated gastric acid secretion to be significantly reduced following RYGB and LSG when compared to the “control” population and the results are summarized in Table 1 [46, 47, 48, 49].

**TABLE 1:** Gastric pH values in patients with obesity and after RYGB and LSG [46, 47, 48, 49]

Study groups	pH (control group)	pH (patients)	Method	Reference
Healthy (*n* = 34); fasting pH	1.5–2.5 (mean 2.0)	N/A	Radiotelemetry capsule (calibrated Heidelberg capsules)	Dressman et al., 1990
**Control group:** lean patients (BMI of 18–25 kg/m^2^) with no comorbid condition; no aspiration prophylaxis **Study group:** BMI ≥ 35 kg/m^2^ and 1 comorbid condition (hypertension); no aspiration prophylaxis; fasting pH	4.3 ± 1.2 (*n* = 20) (mean ± SD)	3.5 ± 1.6 (*n* = 20) (mean ± SD)	pH of gastric aspirate measured intraoperatively using a Cyber scan 510 pH meter (BMI: 41.0 ± 4.6 Kg/m^2^); fasted state	Mahajan et al., 2015
**Control group:** 17 healthy age‐ and gender‐matched control**s** **Study group:** ≥12 months after RYGB	3.65 (1.55) (*n* = 17)	6.45 (0.4) (*n* = 18)	SmartBar (260 kcal, composed of 70% carbohydrate,18% protein, and 3% fat) followed by ingestion of a wireless motility capsule (WMC) Smartpill (ingestible, single‐use capsule) with a total of 200 mL water. PPIs were not administered	Ladebo et al., 2021
Day 1 after LSG	N/A	4.9 ± 1.7 (*n* = 10)	Mean gastric pH post‐LSG; gastric contents aspirated through NG (nasogastric) tube; pH was measured using calibrated pH‐meter. Fasted/fed state not specified	Porat et al., 2021

Animal data for gastric pH is available in a study in which pH changes were assessed using rat models of intact stomach (“sham group”) both following LSG and RYGB [50]. The control group demonstrated no change in gastric pH (2.7 ± 0.47), whereas pH was significantly higher in the LSG (4.7 ± 0.37) and RYGB (4.8 ± 0.50) groups [50]. Based upon the available human and animal studies, a raised gastric pH following RYGB and LSG is therefore to be expected. Postoperatively, patients are also initiated on a PPI (proton pump inhibitor) to prevent gastric ulceration, resulting in a further increase in gastrointestinal pH [51]. The formed pouch post‐RYGB lacks a sphincter and a significant number of parietal cells, responsible for secreting hydrochloric acid into the gastric lumen and therefore more closely resembles small intestinal physiology [49, 52]. Similarly, following LSG, sleeve formation by resection of the greater curvature of the fundus, corpus, and proximal antrum (Figure 1) diminishes gastric acid secretion, but to a lesser extent than RYGB due to the retention of a greater proportion of parietal cells [53].

There is a paucity of published literature on the use of DOACs after bariatric surgery; however, in a single study examining dabigatran concentration after RYGB, a significant reduction in peak serum concentration was demonstrated compared to phase II trial data (34.6 (10–64) ng/mL vs. 184 (64–443) ng/mL) [54]. Dabigatran crucially requires an acidic environment for absorption, demonstrating a decrease in *C*_max_ and AUC of up to 25% in the presence of a raised pH [55, 56, 57]. It is likely therefore that dabigatran, among other drugs, would be significantly affected by pH changes both after RYGB and LSG.

#### 4.1.2 Bile Salts and Phospholipids

Bile salts are critical in enhancing the solubility of drugs with low aqueous solubility, by decreasing surface tension and enhancing wetting [43]. Circulating mean bile acid levels have been found to be significantly lower in obesity compared to the control population (3 μmol/L vs. 7 μmol/L) [58]. Both RYGB and LSG have been shown to permanently change gastrointestinal flora composition, in the absence of long‐term use of proton pump inhibitors, with an increase in *Bacteroides* associated with an increase in biliary acid unconjugation and transformation to secondary bile acids [59]. Compared to RYGB, there is a lack of gastrointestinal manipulation in LSG. Following RYGB, bile flow is increased through the long biliopancreatic limb, isolated from food in the alimentary loop (Figure 1) [60]. It is postulated that it is the presence of the long biliopancreatic limb (Figure 1), that leads to greater changes in biliary acid in RYGB than LSG [61]. Several small studies have examined bile acid concentrations in fasting and postprandial states, both before and after RYGB and LSG. Phospholipids were not examined in the available studies.

Serum concentrations of bile acids have been shown to be higher after RYGB than in patients living with obesity or overweight [62, 63]. A small exploratory study (*n* = 6), thought to be the only one of its kind, examined luminal bile acid concentration (both fasting and postprandial), utilizing catheters positioned to collect intestinal fluids [64]. After RYGB, fasting and post‐prandial total bile acid concentration was found to be 7.5‐fold and 6.5‐fold higher in the common limb (*p* < 0.0001), respectively, compared to patients with obesity before surgery [64]. The results echo similar previous studies, in which bile acid production was shown to increase in both fasting and fed states after RYGB (2.28–4.32 μmol/L [*n* = 12] before surgery vs. 5.38–7.29 μmol/L [*n* = 12] after surgery in a fasted state) [63].

Available findings after LSG are mixed, with some studies indicating a decrease in bile acid synthesis, unchanged, or higher levels after surgery [58, 65, 66]. In one study examining plasma bile acid concentrations, LSG did not significantly change the concentrations, with results remaining close to presurgery levels [58]. However, in a separate study jejunal fasting and postprandial bile acid concentrations, were found to be 3.5‐fold (*p* = 0.009) and 3‐fold (*p* = 0.009) higher compared to obesity after LSG [64].

### 4.2 Gastric Volume

**FIGURE 3:**
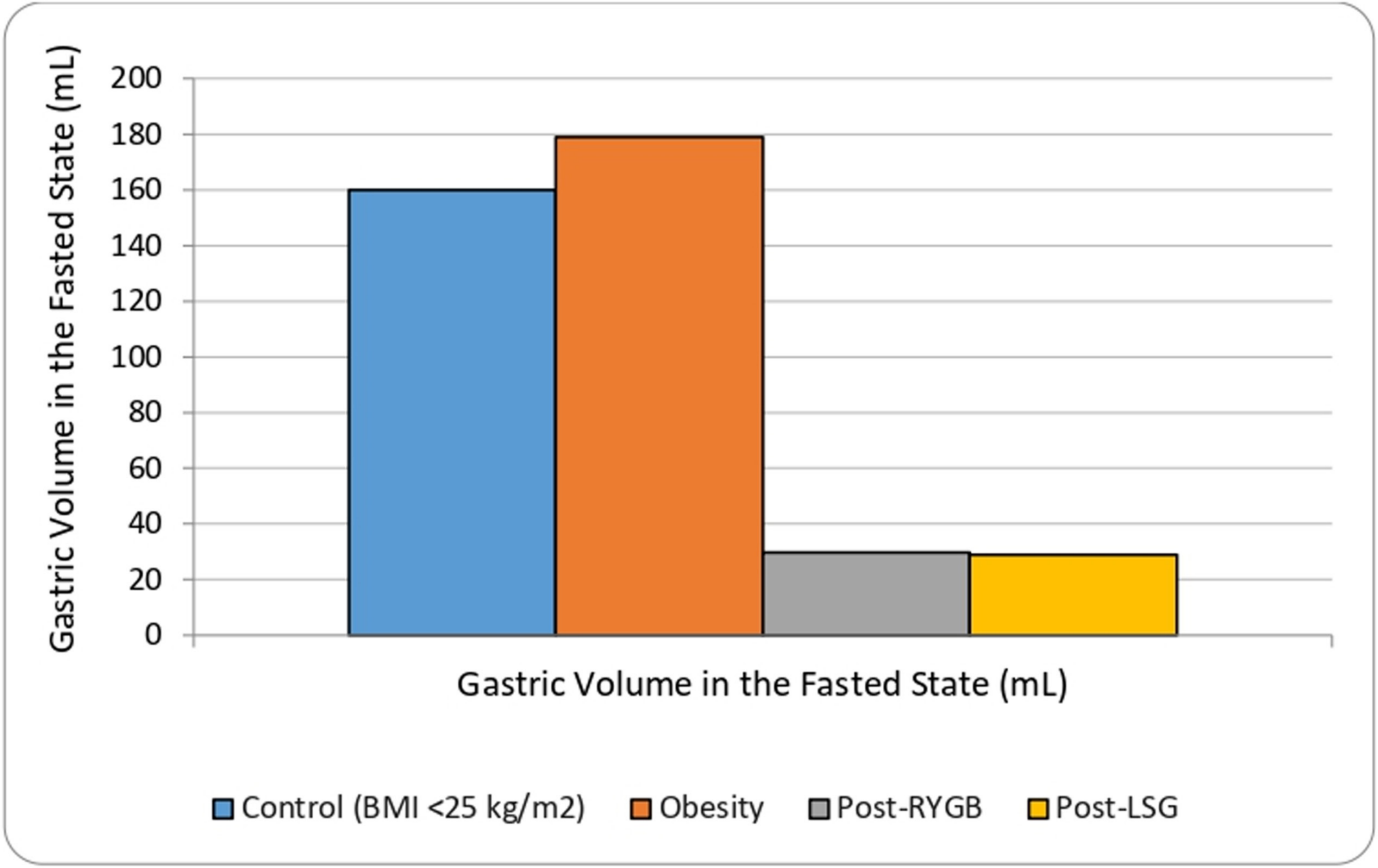
Gastric volume after RYGB and LSG in the fasted state [30, 69, 70].

Reduction in gastric volume has the capacity to significantly reduce drug dissolution and subsequent absorption, particularly of those drugs that are poorly soluble [67]. With reduced gastric capacity, postbariatric surgery patients also often exhibit decreased fluid tolerance, particularly of water, leading to suboptimal fluid intake [68]. Gastric volume in the control population (BMI < 25 kg/m^2^), as depicted in Figure 3, is estimated to be 160 mL in the fasted state [30]. Gastric volume in the population with a BMI ≥ 35 kg/m^2^ (*n* = 38) has been demonstrated via ultrasound assessment to be similar to that in the control population, with a calculated mean of 178.9 mL [69, 71]. Gastric volume values after RYGB, as depicted in Figure 3, are based on median volumes of a population (*n* = 30) after RYGB examined via CT‐volumetry, with values after LSG calculated based on a volume reduction of 83.8% when compared to the control population [62, 70, 71].

### 4.3 Gastric and Intestinal Transit Times

Gastrointestinal transit time is defined as the time taken for luminal contents to empty from the stomach into the small intestine, and as the majority of drugs are primarily absorbed in the small intestine, gastric emptying may be one of the rate‐limiting steps in drug absorption [43]. The significant reduction in gastric surface area following bariatric surgery, when compared to the control population or those living with obesity, may influence gastrointestinal transit time and gastric acid secretion [72].

The impact of bariatric surgery on gastrointestinal transit time has long been recognized, with numerous studies demonstrating accelerated gastric motility (Table 2). It is postulated that gastrointestinal hormonal changes may occur with time after surgery, resulting in the differences seen in the extended follow‐up period. Glucagon‐like peptide 1 (GLP‐1) and peptide YY (PYY) are secreted by the L cells in the gastrointestinal tract, in response to nutrition intake and result in the slowing of gastric emptying [73]. Ghrelin, a motilin‐related peptide and ligand for the growth hormone secretagogue (GHS) receptor, is known to affect gastric emptying by increasing gastrointestinal motility [73, 74].

**TABLE 2:** Gastric emptying time.

Population	Control	Fed/fasted	Result	Method of assessment	Population GET (mins)	Control GET (mins)	% Difference in GET	Reference
Sleeve gastrectomy (3 months postop)	Healthy	Fasted	T_1/2_ gastric emptying	Oral ingestion of the radiotracer sulfur colloid labeled with 99mTc in liquid and solid phase	34.9 ± 24.6 [5–34]	13.6 ± 11.9 [0.12–43]	−61%	Braghetto, 2009
Sleeve gastrectomy (3 months postop)	Healthy	Fed (Egg sandwich)	T_1/2_ gastric emptying	Oral ingestion of the radiotracer sulfur colloid labeled with 99mTc in liquid and solid phase	78 ± 15.01 [66–84]	38.3 ± 18.77 [2–74]	−50.90%	Braghetto, 2009
Sleeve gastrectomy (12 months postop)	Matched with obesity	Fed (2335 KJ)	T_1/2_ gastric emptying	Scintigraphy	106 [calculated]	58 [calculated]	−45.28%	Eiken, 2020
Roux‐en‐Y gastric bypass (12 months postop)	Matched with obesity	Fed (2335 KJ)	T_1/2_ gastric emptying	Scintigraphy	106 [calculated]	71 [calculated]	−33.02%	Eiken, 2020
Roux‐en‐Y gastric bypass (12 months postop)	Presurgery (with obesity)	Fasted	Indirect measure of GE	The time to appearance of D‐xylose in serum, used as an indirect measure of GE	18.6 ± 24.6	7.9 ± 2.7	−57.50%	Wang, 2012

Abbreviations: GE = gastric emptying, GET = gastric emptying time, T_1/2_ gastric emptying = time to reach 50% gastric emptying.

Gastric emptying following LSG at 3 months postoperatively has been effectively demonstrated as accelerated for both solids and liquids, compared to control population controls (BMI 19.8–23.5 kg/m^2^) (34.9 min ± SD 24.6 vs. 13.6 min ± SD 11.9 for liquids and 78 min ± SD 15.01 vs. 38.3 min ± SD 18.77 for solids; *p* < 0.01) [75]. Using paracetamol absorption as a surrogate measure, a small, prospective study (*n* = 9), evaluated subjects at 8 weeks before RYGB surgery and 6 weeks after surgery [76]. The authors demonstrated that liquid emptying occurs more rapidly after RYGB, with the study demonstrating a decreased paracetamol *T*_max_, time to maximum concentration, and 6 weeks after RYGB (19.5 ± SD 6.8 min vs. 6.7 ± SD 2.6 min) [76]. Similarly, in a separate study 1 year after RYGB, the study group (*n* = 7) exhibited an increased rate of gastric emptying with a liquid medium [15]. In an early study in patients randomized to RYGB (*n* = 29), gastric pouch emptying following solid food was found to be slower initially (at 2 months) than a year postoperatively [16]. No statistically significant difference in small‐intestine transit time (*p* = 0.494), colonic transit time (*p* = 0.145), and whole intestine transit time (*p* = 0.145) has been found after RYGB, when compared to controls [77].

In the case of DOACs, apixaban and dabigatran are absorbed primarily in the proximal small intestine [78, 79]. Rivaroxaban however appears to exhibit some gastric absorption, with a 29% decrease in area under the curve (AUC), demonstrated when the drug is released in the proximal small intestine [78]. Because of its poor solubility, rivaroxaban dissolution and subsequent absorption at higher doses are highly dependent on improved solubilization in the presence of food, due to an increase in bile salt concentration [80, 81, 82, 83]. Rivaroxaban *C*_max,_ maximum serum concentration achieved, has been seen to decrease after RYGB in a single‐dose study [84, 85]. Rivaroxaban is a poorly soluble, weak amphoteric compound [86]. However unlike dabigatran, where pH alteration may have a significant impact on oral absorption, other factors, such as gastric transit time and efflux transportation, are thought to have a greater impact on rivaroxaban bioavailability [80, 84, 85].

### 4.4 Metabolizing Enzymes and Intestinal Transporters

RYGB involves the bypass of between 50 and 150 cm of the proximal small intestine [24]. Because of significant removal of the proximal small intestine, the function of the CYP450 enzymes predominantly expressed in this area may be diminished, leading to decreased presystemic intestinal metabolism and alterations in oral drug bioavailability [87, 88, 89, 90]. Oral absorption of drugs occurs via active or passive diffusion depending on the properties of the individual drugs [91]. Active transport, including uptake and efflux transporters (responsible for drug efflux back into the intestinal lumen and resultant reduced absorption), plays a significant role in drug permeation [92]. Abdominal obesity is also associated with elevated levels of pro‐inflammatory cytokines, which are associated with decreased expression of intestinal transport proteins and metabolizing enzymes [93, 94]. Weight loss following surgery may therefore also impact intestinal transport and presystemic metabolism.

Efflux transporter proteins, including P‐glycoprotein (P‐gp), are located on the surface of epithelial cells in numerous sites, and can significantly decrease drug absorption [79, 80, 95]. Using a fexofenadine probe, patients living with obesity have been found to exhibit lower AUC_0–3_ (99.9 ± 98.7), and hence greater P‐gp activity, versus control controls (248 ± 91) (*p* < 0.05) [96]. Although the fexofenadine concentration increased after bariatric surgery (LSG and RYGB) (143.1 ± 174.4), it remained significantly lower than the control group, indicating that P‐gp activity remains higher postoperatively but less than preoperatively [96]. The results of this study support the likelihood that the bioavailability of rivaroxaban and apixaban, substrates for P‐gp, is affected postoperatively [97].

## 5 Other Physiological Parameters Impacting PK

### 5.1 Albumin

A retrospective study of 98 patients after RYGB (*n* = 47) and LSG (*n* = 51) assessed for nutritional deficiencies during 12 months after surgery [98]. When compared to albumin levels in a control population of similarly aged females (41–45 years), no significant difference was observed either with type of procedure or time after surgery [98, 99].

### 5.2 Hepatic Enzymatic Activity

Significant weight loss after bariatric surgery is also thought to influence the activity of drug‐metabolizing enzymes [9]. Hepatic CYP3A4/5 enzyme activity in patients with obesity has been found to be significantly lower before bariatric surgery than in controls (0.419 ± 0.257 vs. 0.633 ± 0.253; *p* < 0.05) [96]. As BMI is seen to decrease, hepatic CYP3A4 activity is seen to increase (1.000 ± 0.590; *p* < 0.05) [96, 100]. In a prospective study in which midazolam (a probe drug for CYP3A4 activity) was utilized to evaluate CYP3A4 activity before and 1‐year after RYGB or LSG, hepatic clearance was 1.7 times higher 1‐year postbariatric surgery [96].

### 5.3 Intestinal Physiology

Levels of tight‐junction proteins in paired jejunal mucosa samples have been determined, at the time of surgery and 6–8 months after RYGB [101]. Decreased mucosal surface and paracellular permeability were noted following RYGB surgery, with altered expression of tight‐junction proteins such as claudins, which are required for the formation and maintenance of tight‐junctions [101].

## 6 Drug‐Related Factors Affecting Absorption After Bariatric Surgery

Bioavailability after oral administration is determined by physiochemical characteristics, including molecular weight (MW) and lipophilicity, with lipophilicity affecting solubility, permeability, and metabolism [102, 103]. Table 3 summarizes the key physiochemical characteristics of drugs commonly prescribed in people living with obesity. As discussed, bile acids are critical in enhancing the solubility of highly lipophilic drugs [43]. Bile acid production is increased after RYGB, with potentially altered bile acid synthesis after LSG [61]. Moderate and low lipophilic drugs (logP < 3), including rivaroxaban, dabigatran, and apixaban (logP 1.5; −2.4 and 2.71, respectively), are less likely to be affected by changes in bile acid concentrations after surgery [89, 113, 120]. Compounds with high lipophilicity, including sertraline and duloxetine require solubilization in bile salt‐phospholipid micelles, to transport the drug to the intestinal wall and therefore are likely to be affected by changes in bile acid production [59, 121, 122].

**TABLE 3:** Drug‐related factors affecting absorption after bariatric surgery for commonly used SSRI/SNRIs and DOACs.

Drug	LogP	pKa	Behavior at altered physiological pH	Molecular weight (g/mol)	Formulation	BCS class
Sertraline	4.30 ± 0.01 [104]	9.48 [104]	Likely to exhibit decreased solubility [50, 51, 77]	343 [105]	Immediate‐release tablets	II [106]
Citalopram	3.58 [107]	9.5 [107]	Likely to exhibit decreased solubility [50, 51, 77]	405.35 [108]	Immediate‐release tablets	I [109]
Venlafaxine	3.20 [110]	8.92 [111]	Likely to exhibit decreased solubility [50, 51, 77]	277.4 [112]	Immediate‐release tablets	I [111]
Apixaban	2.71 [113]	13.2 [114]	Likely to exhibit decreased solubility [50, 51, 77]	459 [115]	Immediate‐release tablets	III [115]
Rivaroxaban	1.5 [89]	−1.6; 13.4 [86, 116]	Unlikely to be impacted significantly [80, 84, 85]	436 [117]	Immediate‐release tablets	II [89]
Dabigatran	−2.4 [113]	4.0 [118]	Likely to exhibit decreased solubility [55, 56, 57]	472 [119]	Immediate‐release tablets	II [78]

Molecules with a MW less than 350 g/mol and low lipophilicity can permeate through tight junctions via paracellular transport [123, 124]. As discussed, altered post‐RYGB expressions of claudins, which are required for the formation and maintenance of tight junctions, have the potential to contribute to decreased uptake of hydrophilic drugs of low MW [101].

**FIGURE 4:**
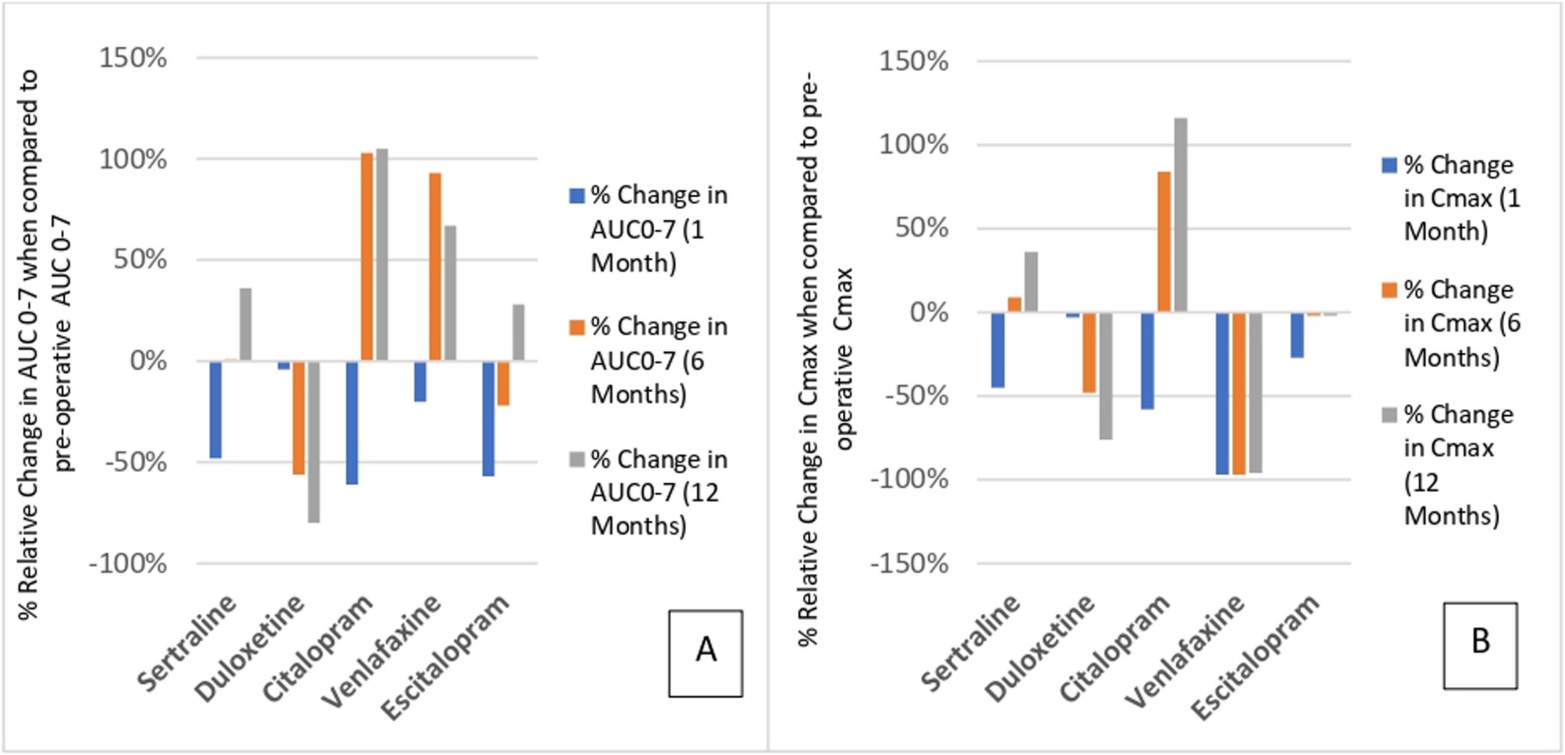
(A) % Relative effect in AUC_0–7_ at 1, 6, and 12 months after RYGB [104, 125, 126, 127, 128]. (B) % Relative effect in *C*_max_ at 1, 6, and 12 months after RYGB [104, 125, 126, 127, 128], of commonly used SSRI/SNRIs.

Case reports highlight individual cases of SRNI/SRRI therapeutic failure after RYGB surgery, including a decrease of 64% in the postoperative bioavailability of sertraline, accompanied by a relapse of symptoms of anxiety [125]. Figure 4A depicts the % relative change in AUC_0–7 h_ of commonly used SSRI/SNRIs at 1, 6, and 12 months post‐RYGB when compared to preoperative AUC_0–7 h_ [104, 125, 126, 127, 128], with Figure 4B depicting the % relative change in *C*_max_ (maximum concentration) at 1, 6, and 12 months after RYGB [104, 125, 126, 127, 128].

Multiple physiological changes have been demonstrated to occur with time since surgery. The appearance of the intestinal mucosa has also been shown to be altered 6–8 months after RYGB surgery, with the height of villi decreased and associated reduction in resultant surface area [110]. BCS Class I compounds are readily soluble and are thought to saturate efflux transporters, whereas Class II compounds, such as duloxetine, are bound by their low solubility and are unable to effectively bypass the effects of the efflux transporters [92]. This may account for the alteration in duloxetine decrease in AUC_0–7_ and *C*_max_, with time from RYGB surgery.

The degree of ionization is a highly significant factor in overall drug absorption and is dependent on the pH of gastrointestinal contents, as well as the acidic or basic nature of the drug itself [129]. As a result of the raised GI pH following RYGB and LSG, drugs that are weakly basic in nature, for example, venlafaxine, sertraline, citalopram, and apixaban, will demonstrate decreased solubility due to less ionization [50, 51, 77]. Both rivaroxaban (BCS Class II) and apixaban (BCS Class III) are substrates for efflux transporter proteins P‐gp and breast cancer resistant protect (BRCP), which are located on the surface of epithelial cells in numerous sites, including in the gastrointestinal tract, with dabigatran etexilate (BCS Class II) having a moderate affinity for P‐gp [79, 80, 95].

With numerous factors affecting drug bioavailability, the clinical impact of potential differences in absorption is currently unable to be easily quantified using available data. The peri‐operative management of pharmacotherapy is typically planned as part of the preoperative process. Table 4 proposes an overall risk stratification based on physiochemical characteristics and the best available evidence, to be considered when managing pharmacotherapy prior to RYGB or LSG in the absence of relevant pharmacokinetic data for specific drugs. The bioavailability of BCS class I drugs, with high aqueous solubility and high permeability, is unlikely to be affected by RYGB or LSG, with BCS class II drugs likely to be affected to a greater extent. As previously discussed in this review, decreased mucosal surface and paracellular permeability have been noted following RYGB surgery, with no available data after LSG [101]. With low permeability, BCS Class III and IV drugs could be affected by changes in membrane permeation postoperatively [8, 9].

**TABLE 4:** Summary of risk stratification to be considered when managing pharmacotherapy prior to RYGB or LSG in the absence of relevant pharmacokinetic data for specific drugs.

Risk category	Characteristics	Action	References
Low risk	High aqueous solubility and high permeability (BCS Class I)	Available evidence supports prescribing post‐RYGB/LSG	[104, 125, 126, 127, 128]
Medium risk	Low % ionization	Available evidence supports prescribing with close monitoring	[104, 125, 130, 131, 132]
High risk	Low aqueous solubility and high permeability (BCS Class II); low permeability (BCS Class III and IV)	Prescribing is not advised in light of available evidence	[104, 125, 126, 127, 128]

## 7 Formulation Related Factors Affecting Absorption After Bariatric Surgery

Because of a restrictive liquid diet, modified release preparations are often switched to immediate release preparations following bariatric surgery [133]. In addition, tablets may be crushed and capsules opened before administration in the initial postoperative phase [133]. The impact of such dosage form manipulation is currently unknown. However, there are numerous potential risks, in particular variation in bioavailability, therapeutic efficacy, and an increased risk of adverse drug reactions [134].

Dissolution of crushed antidepressants, anxiolytics and antipsychotic immediate release tablets was examined after RYBG using an in vitro model, compared to a control in which uncrushed tablets were added to a pseudo‐gastric medium [133]. The dissolution media used in the study were lactated Ringer's at 37°C, with the pH after RYGB (6.8) and control (1.2) produced by the addition of hydrochloric acid (control) or sodium bicarbonate (post‐RYGB model) [133]. The study did not incorporate a simulation of bile or pancreatic secretions but links to other physiological changes seen after bariatric surgery, with a significant reduction in gastric acid secretion following RYGB and LSG when compared to the “control” population [46, 47, 48, 49, 133].

Citalopram and venlafaxine exhibited unchanged dissolution of crushed tablets in the in vitro RYGB model, compared with decreased dissolution of the crushed sertraline tablet, where a median 30 mg of the 100‐mg tablet dissolved postoperatively, compared to 50 mg preoperatively (*p* < 0.05) [133]. The decreased dissolution of sertraline tablets in the post‐RYGB medium (with a raised pH) was demonstrated despite the increase in surface area with the change in formulation to crushed tablets. Unlike BCS class I drugs such as citalopram and venlafaxine, the rate‐limiting step in the absorption of BCS class II drugs, such as sertraline, having low water solubility and high permeability, is most likely the low solubility/poor dissolution [125, 127].

## 8 Physiological‐Based Pharmacokinetic Modeling

Physiological‐based pharmacokinetic (PBPK) modeling combines knowledge of drug and formulation properties with human physiological data (control and disease state) to produce a computational prediction of drug plasma concentration‐time profiles [135]. PBPK modeling has key advantages over traditional PK approaches. PBPK modeling is able to predict the in vivo performance for a population of individuals in whom consistent clinical information is lacking, while incorporating multiple pathophysiological factors, not easily achieved using a traditional PK approach [136, 137]. The use of PBPK modeling, particularly considering recent progress in defining physiological parameters after bariatric surgery, would enable a mechanistic understanding of the absorption process to be obtained and a more thorough assessment of any risk associated with their therapeutic outcomes.

## 9 PBPK Modeling After Bariatric Surgery: Current Status

Several papers have previously been published on PBPK modeling after bariatric surgery (including LSG, RYGB, and postbiliopancreatic diversion with duodenal switch), including most notably, a virtual model of five drugs (omeprazole, diclofenac, fluconazole, ciprofloxacin, and simvastatin) [138]. The model integrated changes in numerous parameters including GI pH, gastric volume, gastric emptying, and renal function. The modeling was limited at the time by the lack of clinical data and the lack of data regarding physiological changes postoperatively, with aspects such as changes in small intestinal transit time subsequently found to be nonsignificant [77, 139].

Postoperative anatomical changes and aspects such as the impact of the VLCD have also not been previously considered in PBPK modeling. Findings were mixed but with definite trends towards changes in oral bioavailability being dependent on both the surgical procedure and specific drug parameters, including solubility and permeability [138].

Recently physiologically based pharmacokinetic‐pharmacodynamic (PBPK/PD) modeling was utilized to examine the effect of obesity and RYGB surgery on omeprazole PK [140]. Upregulation of hepatic CYP3A4 activity, following bariatric surgery was employed as part of the model, to predict the effect of obesity and RYGB on omeprazole PK and intragastric pH [140]. Sensitivity analysis demonstrated that hepatic CYP3A4 and CYP2C19 have a significant impact on omeprazole AUC [140]. The authors' findings indicate a higher intragastric pH than the control or presurgery population with obesity [140]. Similarly to other attempts at PBPK modeling, only RYGB was considered. Interestingly, small intestinal transit time was reduced in the model to 3.5 h; however, changes in small intestinal transit time have been recently found to be nonsignificant [77, 139, 140].

## 10 Population Characteristics

PBPK models in special populations, such as those after bariatric surgery, are built from aggregation of prior knowledge, coupled with the use of calculations, to produce an understanding of physiological differences between control adult reference populations and the populations being examined [135]. Table 5 includes a summary of our proposed key physiological parameters for new populations of patients with obesity (pre‐ and post‐VLCD) and following RYGB and LSG (at 3, 6, and 12 months after surgery) [15, 47, 48, 49, 69, 70, 71, 75, 77, 98, 100, 139]. We proposed these physiological parameters based on our literature review, as outlined in the sections of this review on gastric volume, gastric and intestinal transit times, gastric fluid composition, albumin, and hepatic enzyme activity.

**TABLE 5:** Summary of physiological parameters in obesity, after VLCD and after RYGB and LSG (at 3, 6, and 12 months after surgery).

Presurgery to postsurgery physiological parameters	Surgery	Obesity	After VLCD	3 months after surgery	6 months after surgery	12 months after surgery	References
Gastric emptying of liquids (min)	RYGB	18.6	18.6	7.9	7.9	7.9	[15]
	LSG	18.6	18.6	13.6	13.6	13.6	[75]
Gastric volume (mL)	RYGB	178.9	178.9	29.75	29.75	29.75	[70, 71]
	LSG	178.9	178.9	28.98	28.98	28.98	[69, 71]
Gastric pH (fed)	RYGB	—	—	6.45	6.45	6.45	[49]
Gastric pH (fasted)	LSG	3.5	3.5	4.9	4.9	4.9	[47, 48]
Small intestinal transit time (h)	RYGB and LSG	Unchanged from “healthy” (fasted state) [77, 139]					
Human serum albumin levels (g/L)	RYGB	41.0	41.0	39.8	39.8	39.8	[98]
	LSG	41.0	41.0	41.3	41.3	41.3	[98]
Hepatic CYP3A4 (metabolic ratio)	RYGB	3.38	—	2.29	2.29	2.29	[100]

## 11 Limitations

There is limited research in physiological parameters affecting pharmacokinetics in obesity and after RYGB and LSG surgery. There are no studies exploring cardiac volume after RYGB and LSG. Because of this lack of data, mean heart weights in relation to decreasing BMI were determined from a large study of autopsies, with myocardial tissue density used to calculate heart volume. Further clinical studies are also needed measuring serum drug concentrations in patients after RYGB and after LSG, with any changes in concentration over time after surgery also captured.

## 12 Conclusions

With numerous factors affecting drug bioavailability, the clinical impact of potential differences in absorption cannot be easily quantified using current data and further understanding of the effects of surgery on bioavailability is therefore urgently needed to support safer care. Anatomic changes, unexamined in previous studies, including alterations in hepatic and renal volume, are expected to have a significant impact on renal and hepatic clearance. As SSRIs/SNRIs are extensively hepatically metabolized, their clearance is likely to be affected by the altered liver size, with DOACs that are reliant to some extent on renal clearance likely to be less affected.

Because it is currently not possible to predict which patients are at the highest risk of experiencing the clinical effects of toxicity or therapeutic failure, it is recommended that dose adjustments are not made pre‐emptively. A clearer understanding of the effect of physiological alterations and weight loss on drug performance after surgery would support more evidence‐based medicine optimization in this patient group.

## Author Contributions

DW conceptualized the review, performed the literature searches, and wrote the original draft. DW, AE, NF, and MJ all contributed to reviewing and editing the final manuscript.

## Funding

This work was supported by the Pharmacy Research UK (PRUK‐2021‐GA3‐DW).

## Conflicts of Interest

The authors declare no conflicts of interest.

## Data Availability

Data sharing not applicable to this article as no datasets were generated or analysed during the current study.
